# Neuroplasticity and Multilevel System of Connections Determine the Integrative Role of Nucleus Accumbens in the Brain Reward System

**DOI:** 10.3390/ijms22189806

**Published:** 2021-09-10

**Authors:** Martyna Bayassi-Jakowicka, Grazyna Lietzau, Ewelina Czuba, Aleksandra Steliga, Monika Waśkow, Przemysław Kowiański

**Affiliations:** 1 Department of Anatomy and Neurobiology, Faculty of Medicine, Medical University of Gdańsk, 1 Dębinki Str., 80-211 Gdańsk, Poland; mbayassi@gumed.edu.pl (M.B.-J.); ewelina.czuba@gumed.edu.pl (E.C.); 2 Karolinska Institutet, Department of Clinical Science and Education, Södersjukhuset, Internal Medicine, Sjukhusbacken 10, 118 83 Stockholm, Sweden; 3 Institute of Health Sciences, Pomeranian University of Słupsk, 64 Bohaterów Westerplatte Str., 76-200 Słupsk, Poland; aleksandra.steliga@gmail.com (A.S.); monikawaskow@gmail.com (M.W.)

**Keywords:** addiction, brain reward system, neurodegeneration, neuroplasticity, nucleus accumbens

## Abstract

A growing body of evidence suggests that nucleus accumbens (NAc) plays a significant role not only in the physiological processes associated with reward and satisfaction but also in many diseases of the central nervous system. Summary of the current state of knowledge on the morphological and functional basis of such a diverse function of this structure may be a good starting point for further basic and clinical research. The NAc is a part of the brain reward system (BRS) characterized by multilevel organization, extensive connections, and several neurotransmitter systems. The unique role of NAc in the BRS is a result of: (1) hierarchical connections with the other brain areas, (2) a well-developed morphological and functional plasticity regulating short- and long-term synaptic potentiation and signalling pathways, (3) cooperation among several neurotransmitter systems, and (4) a supportive role of neuroglia involved in both physiological and pathological processes. Understanding the complex function of NAc is possible by combining the results of morphological studies with molecular, genetic, and behavioral data. In this review, we present the current views on the NAc function in physiological conditions, emphasizing the role of its connections, neuroplasticity processes, and neurotransmitter systems.

## 1. Structure and Function of Nucleus Accumbens

### 1.1. Localization of Nucleus Accumbens on the Border between Motor and Limbic Areas Suggests Its Integrative Role in the Brain Reward System

**Figure 1:**
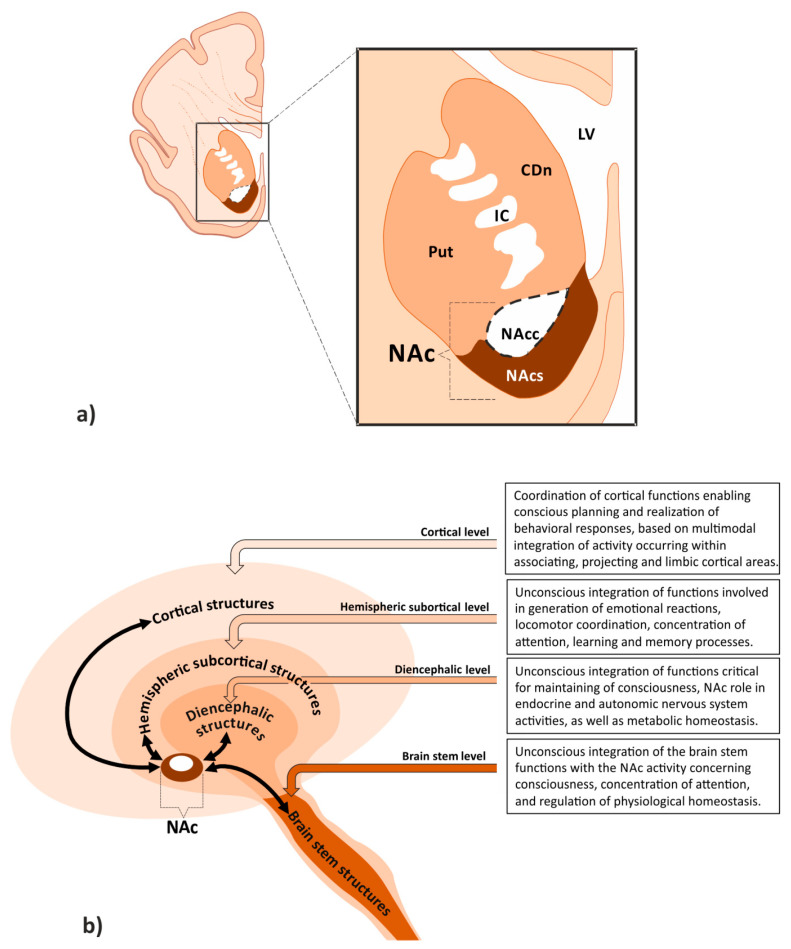
**Nucleus accumbens localization and connections**. (**a**) Coronal cross-section through the human brain at the level of nucleus accumbens (NAc) occupying the lowest part of striatum, poorly separated from the other areas and divided into a centrally localized core (NAcc) and a peripherally situated shell (NAcs). Medially and upwards, it changes into the caudate nucleus (CDn), and laterally into the putamen (Put). Whereas the dorsal part of the striatum belongs to the locomotor system, its ventral part is considered as a structure of the limbic system. Fibers of the internal capsule (IC) surround NAc superiorly. The localization of NAc on the border of motor and limbic structures enables its integrative and coordinating function upon both systems. (**b**) Due to its localization in the center of the hemisphere, NAc can develop extensive connections with numerous brain areas that can be divided into structural and functional levels (i.e., cortical structures, hemispheric subcortical structures, diencephalic structures, and brain stem structures). This hierarchical arrangement reflects not only the consecutive stages of phylogenetic brain development but also the pattern of cooperation among several functional systems representing various levels of integration.

A group of morphologically and functionally related brain structures receiving and interpreting stimuli associated with satisfaction, positive feeling, and addiction is commonly defined as the brain reward system (BRS). This system consists of subcortical mesolimbic structures, such as the nucleus accumbens (NAc), ventral tegmental area (VTA), amygdala (Amg), striatum (Str), and septum (Spt). It also includes several meso-corticolimbic regions, such as the hippocampus (Hip), prefrontal cortex (PFC), para-hippocampal, entorhinal, and motor function-related cortical areas [1]. NAc is regarded as one of the most important elements of the BRS [2]. It occupies the ventral part of the brain hemisphere (Figure 1a). Situated below the internal capsule, NAc extends beneath its anterior crus posteriorly, until the end of anterior commissure. Without clear boundaries, it goes into two areas of the motor system: the putamen (Put) laterally, and the caudate nucleus (CDn) medially. Together with the olfactory tubercle, the NAc is included in the ventral Str, being a part of the limbic system [3]. Localization of the NAc determines its integrative role within the BRS and involvement, along with other structures, in physiological processes. Difficulties in precisely defining NAc borders using classical morphological methods justify further studies employing neuroradiological imaging techniques, which could allow for objective assessment of its volume. Based on morphological and immunohistochemical studies, as well as on the analysis of its connections, two parts of the NAc have been distinguished: the shell and the core [4,5,6].

### 1.2. Morphological and Molecular Characteristics of the Accumbal Neurons Determine Their Integrative Role in the Brain Reward System

NAc contains predominantly small and medium-sized spiny neurons (MSNs) [7,8]. Among them, the GABA-ergic neurons with dopaminergic and glutamatergic synapses on dendritic spines are the most numerous [9,10]. Characteristic differences have been found in cellular structure between the shell and core [8,11]. These concern not only the shape and neuronal density, but also the morphology of dendritic trees and spines. In humans, neurons are more densely distributed in the shell than in the core [7]. These are predominantly fusiform and multipolar cells, with well-developed dendritic trees, visible second- and third-order branches, and numerous dendritic spines. In the core, the most numerous are pyramidal and multipolar neurons, with clearly visible spines on their secondary branches. Interspecies differences concerning neuronal size and number of dendritic tree branches in the two parts of NAc have also been reported [8,9,11].

Each part of NAc, namely shell and core, has different molecular characteristics considering the type of released neurotransmitters and their receptors. Levels of neurotransmitters, such as dopamine (DA), serotonin (5-HT), and norepinephrine (NE) are different in the two parts of NAc [12,13]. While the basal concentration of NE is higher in the shell than in the core in resting conditions, DA basal concentration in the core is twice as high as in the shell [14]. After stimulation with amphetamine, the increase in concentration of both neurotransmitters has been observed in both parts of NAc. However, the increase of NE is higher and lasts longer in the shell compared to the core, while DA concentration is higher in the core and lasts equally long in both parts of the nucleus. These observations suggest a functional differentiation between the two parts of NAc. Different morphological adaptations to turnover of NE and DA, as well as variations in neuronal sensitivity to the action of amphetamine can explain the differences in both neurotransmitters’ concentration observed in NAc shell and core. The category of morphological adaptations includes a difference in the innervation density of both parts of NAc by noradrenergic and dopaminergic projections, as well as differences in the structure of synapses and density of transporters representing both neurotransmitter systems [15]. Functional adaptations include a different sensitivity of neurotransmitter transporters to the effects of amphetamine, which is associated with reuptake-blocking and neurotransmitter release from vesicular pools in the NAc shell and core [16,17]. Moreover, due to the interaction of both neurotransmitter systems, the amphetamine action upon dopamine transporters may cause changes in the concentration of not only DA but also NE in the NAc shell and core [18].

In humans, the concentration of NE is different in various portions of NAc. Tong et al. reported three-fold higher concentration of this neurotransmitter in the caudomedial portion than in its caudolateral part, and 12-fold higher in the caudal fragment compared to the rostral [18]. DA levels in NAc are more evenly distributed in an antero-posterior direction, demonstrating a decreasing trend towards its caudal fragment. The caudomedial portion of the human NAc contains equally high concentrations of NE and DA, probably the only area in the brain where the levels of both these transmitters are comparable [18]. Interestingly, the comparable, high concentrations of NE and DA in the caudomedial portion of NAc have not been reported in other mammalian species, such as the rat [19], rabbit [20] or non-human primates [21], in which the NE content does not exceed 20% of the DA concentration [18]. So far, the functional significance of these apparent evolutionary differences has not been elucidated.

Apart from the internal functional specialization within NAc, high concentrations of NE and DA in the same area may suggest an interrelationship between both neurotransmitter systems and their reciprocal modulatory function upon controlled processes. On the other hand, the 5-HT content is higher in the shell compared to the core, although its utilization is greater in the latter [12]. High levels of γ-aminobutyric acid (GABA) and glutamate (Glu) are present in both parts of NAc [9,22]. Apart from neurotransmitters, neuropeptides, proteins, and some types of receptors also have characteristic distribution patterns within NAc. Relatively large amounts of substance P and calretinin are found in the shell [23,24], while calbindin [24], enkefalin [24,25], and GABA receptors [26] are present mainly in the core.

The degree of morphological differentiation of neurons in NAc, their molecular characteristics, as well as the development of their spino-dendritic system determine the optimal adaptation of this brain structure to the integrative function which it plays within the BRS.

### 1.3. Nucleus Accumbens Connections Enable Coordination of Limbic, Motor and Vegetative Functions

A functionally important feature of NAc is its extensive system of connections with numerous brain areas. These can be divided into structural levels of cortical, hemispheric subcortical, diencephalic, and brain stem areas (Figure 1b). This hierarchical schedule of connections is useful not only for understanding the phylogenetic sequence of brain development [27,28,29] but also for explanation of the cooperation patterns and various levels of integration between the functional systems supporting the NAc activity within the BRS.

**Figure 2:**
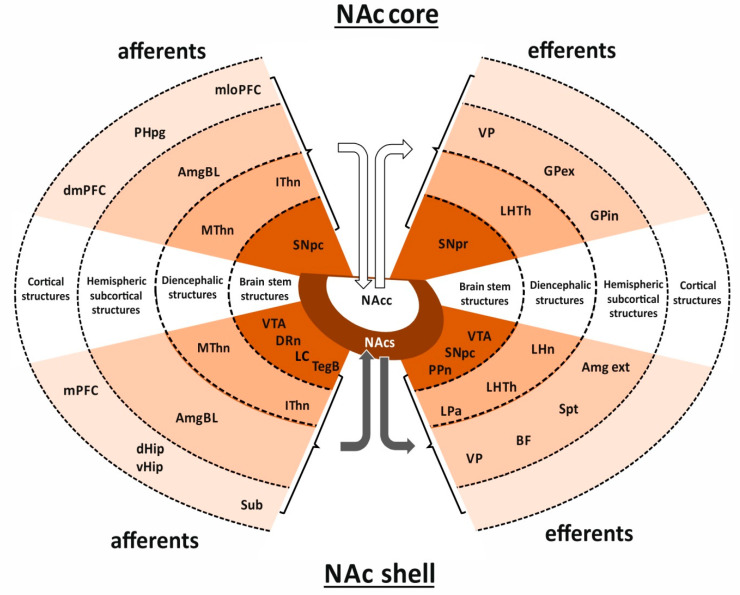
**Hierarchical pattern of connections in the nucleus accumbens’ core and shell**. There are significant differences between the NAcc and NAcs afferent and efferent connections. While afferent projections to NAcc originate from all structural levels i.e., cortical, hemispheric subcortical, diencephalic, and brain stem areas, its efferent projections mainly reach the subcortical brain structures and the brain stem. Afferent and efferent NAcs projections are more widely distributed and come from/to all brain structural levels. Furthermore, to a large extent they comprise other structures than those related to NAcc. This emphasizes the structural and functional differentiation between the two parts of NAc. The core of NAc receives afferents from the cortical limbic areas, such as the dorso-medial PFC (dmPFC; dorsal prelimbic, anterior cingulate cortex), medial and lateral orbital PFC (mloPFC), and para-hippocampal gyrus (PHpg). This part of the BRS also receives projections from the anterior part of AmgBL, as well as from the thalamic nuclei associated with nonspecific cortical stimulation (e.g., MThn and IThn). Finally, it is also a target of the brain stem projections originating from the motor system-related area in SNpc.

Significant differences in the topography of connections between the two parts of NAc can be explained by their functional diversity (Figure 2) [2,3,4]. The shell receives projections originating from the cortical areas of the limbic system, such as the medial prefrontal cortex (mPFC; i.e., infralimbic and ventral prelimbic cortex), subiculum (Sub), as well as the dorsal and ventral hippocampus (dHip and vHip, respectively). In addition, this part of NAc receives projections from subcortical limbic structures, such as the basolateral part of Amg (AmgBL) and the midline and intralaminar thalamic nuclei (MThn and IThn, respectively). Functionally important projections from the brain stem centers, such as VTA, dorsal raphe nucleus (DRn), locus coeruleus (LC), and bulbar tegmentum (TegB) also terminate in the shell (afferent projections) [4,6,30,31]. A characteristic feature of these projections is the involvement of various neurotransmitters, such as DA, Glu, GABA, 5-HT, and NE.

The target areas of projections originating from the shell are limbic structures, such as extended Amg (Amgex), Spt, basal forebrain (BF), ventral pallidum (VP), and diencephalic areas involved in the regulation of vegetative and limbic functions, such as lateral preoptic area (LPa), lateral hypothalamus (LHTh), and lateral habenular nucleus (LHn) [2,3,4]. The NAc shell also projects to the brain stem areas involved in motor functions, such as pars compacta of substantia nigra (SNpc), structures of the BRS, such as VTA or those involved in the activation of diencephalic and telencephalic regions responsible for maintenance of consciousness, attention and learning, like pedunculopontine nucleus (PPn) [32,33,34,35]. The similarities between the efferent projections of the shell and Amg are at the basis of the hypothesis that the shell is a transitional zone between Str and Amg [36]. Through the indirect pathway via VP and the mediodorsal thalamic nucleus (MDThn), projections from the shell influence VTA and the PFC. This results in alterations in DA release and, consequently, its effects exerted upon meso-cortical areas related to the reward mechanisms [4,37,38]. Finally, projections from the shell are also involved in the function of the motor system [39,40]. The NAc connections to cortical areas related to motor function, such as motor cortex (Mctx) and premotor cortex (preMctx), are not direct. On their way to these cortical areas, the impulses pass through the subcortical structures, such as VP, SNpc, and thalamic nuclei (e.g., MDThn) [3,4,6]. This pattern of connections within the basal ganglia-cortico-thalamic loop enables integration of signals of different origins. In contrast to the cortical projections from the dorsal part of striatum, the projection from its ventral part is more dispersed [39,40].

The NAc core is a source of efferent projections targeting areas of the basal ganglia related to the limbic and motor systems, e.g., VP, external and internal parts of globus pallidus (GPex and GPin, respectively). Neurons located in the core also project to pars reticulata of substantia nigra (SNpr) [4,6,30].

**Figure 3:**
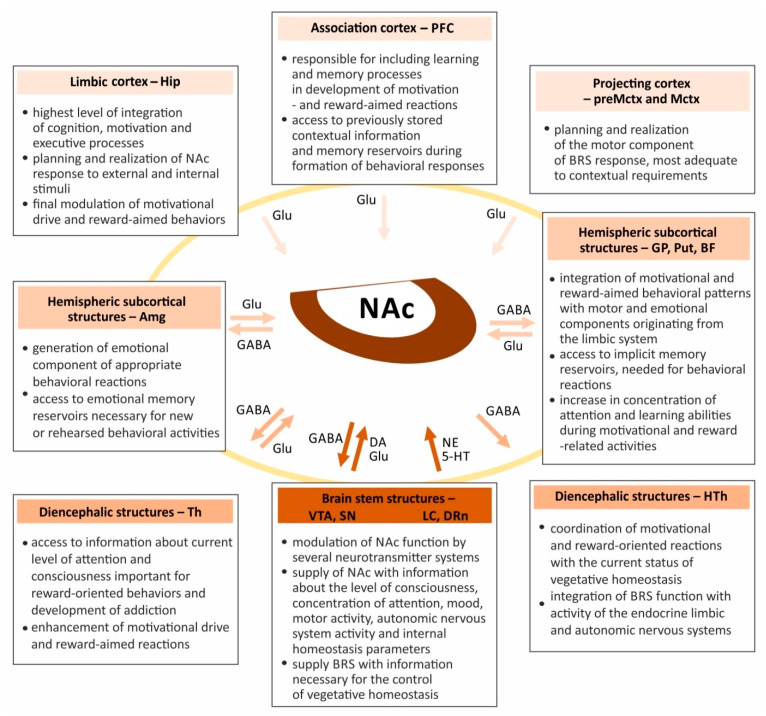
**Functional significance of the nucleus accumbens connections**. The hierarchical arrangement of NAc connections with cortical, hemispheric subcortical, diencephalic, and brain stem structures illustrates its involvement in the activation of different functional systems and explains the NAc’s integrative and coordinative role on different functional levels. NAc has connections with the fields of association, projection and limbic cortices. This enables conscious and precise planning of behavioral activity based on integration of multimodal information (association cortex), along with planned and consciously performed locomotor activity (projecting cortex), and information coming from the declarative (explicit) memory reservoirs (limbic cortex). Afferent connections of NAc with the hemispheric subcortical structures transfer information from the areas responsible for creating emotional reactions (Amg), locomotor coordination (GP and Put), and from those related to increase in the concentration of attention and learning ability (BF). They also allow the use of data stored in the reservoirs of emotional and procedural (implicit) memory (Amg and basal nuclei, respectively). Afferent connections of NAc with the diencephalon structures, on the one hand, allow the inflow of information about the current state of consciousness and concentration of attention (Th). They allow coordination of the NAc’s activities with the endocrine system, activation of the autonomic nervous system, and metabolic processes (HTh). The NAc’s connections with the brain stem structures, due to the involvement of several neurotransmitter systems, ensure the precision of their regulation and functioning. These connections allow transfer of information about the state of consciousness and attention (VTA and LC), motor activity (SN), mood (DRn), and physiological homeostasis (LC). The multilevel system of the NAc connections with the other brain areas is based on a dynamic balance between the stimulatory and inhibitory effects of numerous neurotransmitters and modulators.

Altogether, both parts of NAc have extensive ascending and descending connections which allow this nucleus activity to be coordinated with several cortical areas related to the association, limbic and projection functions (Figure 3) [27]. This enables conscious and precise planning of behavioral activity based on an integration of multimodal information (association cortex) along with a planned and consciously performed locomotor activity (projecting cortex), and information coming from the declarative (explicit) memory reservoirs (limbic cortex) [41]. Afferent connections of NAc with the hemispheric subcortical structures transfer information from the areas responsible for creating emotional reactions (Amg), locomotor coordination (GP and Put), and from those related to increase in the concentration of attention and learning ability (BF) [4,6,30]. They also allow the use of data stored in the reservoirs of emotional and procedural (implicit) memory (Amg and basal nuclei, respectively) [36]. Additionally, NAc connects with several diencephalic structures that allows the inflow of information about the current state of consciousness and concentration of attention (Th). This enables coordination of the NAc’s activities with the endocrine system, activation of the autonomic nervous system, and metabolic processes (HTh). Finally, connections of NAc with the brain stem structures, due to the involvement of several neurotransmitter systems, ensure the precision of their regulation and functioning [4,6,30,31]. These connections allow transfer of information about the state of consciousness and attention (VTA and LC), motor activity (SN), mood (DRn), and physiological homeostasis (LC).

### 1.4. Integrative Role of the Nucleus Accumbens Requires Cooperation of Several Neurotransmitter Systems and Receptors, Which Modulate Synaptic Plasticity and Determine the Effects of Drugs on Behavioral Responses

The role of NAc as an important integrating center in the elaboration of behavioral reactions of the limbic, motor and vegetative systems is possible due to projection pathways using several neurotransmitters, of which DA, Glu and GABA could be considered as of primary importance, although serotoninergic and noradrenergic projections also contribute to the specific NAc functions.

Numerous studies indicate a crucial role of receptors representing all the main neurotransmitter systems in physiological and pathological processes in NAc [2,42,43,44]. Their function is based on the initiation not only of the molecular processes of synaptic plasticity but also morphological changes in the spino-dendritic system, being the base for development of short- and long-term synaptic plasticity processes.

#### 1.4.1. Dopaminergic System

The sources of dopaminergic projections that reach the NAc shell and core are VTA and SN, two mesencephalic structures involved in functioning of the limbic and motor systems, respectively [45,46,47]. While the projections coming from VTA terminate mainly in the shell, the target of the SNpc-originating projections is primarily the core [48,49,50]. This suggests that the shell interacts with the mesolimbic system, and the core with the nigrostriatal [42,51].

The DA released in NAc affects many physiological processes. The level of this neurotransmitter increases in this brain area with the reward approaching, which reflects the awaiting state for its achievement. DA in NAc has also a positive effect on motivation for reward-achievement behaviors and reward-driven learning. Finally, it enhances learning of prediction errors, important in planning new adaptive behaviors [52]. Regulation of DA release is a complex process which depends on the cause of activation and the goal to be achieved, as well as on origin of the activated projection. Consequently, there is a specific impulse-like pattern of DA release in learning and motivational activities [53]. The manner in which DA is released could determine the type of activated behavioral response. Recent studies have shown that the release of DA in the core of NAc can be a signal to focus attention on behaviors aimed at reward achievement, although it does not have to be a direct consequence of VTA neuronal stimulation [53,54,55]. Therefore, there is no simple relationship between the stimulation of dopaminergic neurons in VTA and the amount of DA released in NAc. The complicated regulation of DA release allows for increased precision in controlling the NAc functions.

The modifying effect of DA on synaptic plasticity is based on changes in receptors’ activation and on the stimulating or inhibiting character of their response, changing the probability of neurotransmitter release and cell excitability, as well as on triggering synaptic potentiation or depression [56]. Located in the GABA-ergic projecting MSNs, dopaminergic D1 and D2 receptors (D1R and D2R) exert stimulatory and inhibitory effects, respectively [48]. Whereas projections of GABA-ergic neurons localized in the dorsal striatum form well-defined direct and indirect pathways, the GABA-ergic projections originating from NAc are not so clearly distinguished [42]. It has been suggested that the target area for D1R-containing MSN projections is primarily VTA, while both D1R- and D2R-containing MSNs project to VP [57,58]. Poorly defined projection targets, together with different mechanisms of synaptic plasticity in both subpopulations of neurons, could explain their different functions in behavioral responses in both physiological and pathological conditions.

One of the most important functions of DA in NAc is its modulatory effect on the processes of short- and long-term homeostatic synaptic plasticity [59,60]. However, it often requires an interaction with other neurotransmitter systems, such as glutamatergic, noradrenergic, and serotonergic [2,56,61]. The modulatory effect of DA is based on regulation of the amount of neurotransmitters released in the area of dendritic spines [62], receptor externalization [59], and trafficking of AMPAR, GluA1, NMDAR receptors [59,63,64]. All these processes occurring in the BRS require involvement and cooperation among receptors representing all major neurotransmitter systems [56].

Apart from the essential physiological functions, the dopaminergic system plays fundamental role in development of addiction [65] which is the result of interaction of several factors, such as the influence of the environment, internal factors such as metabolic and genetic conditions, coexisting diseases, as well as pharmacokinetic and pharmacological properties of the drug itself [66]. According to the most recent hypothesis, an involvement of the dopaminergic mesolimbic system best explains the pathophysiological effects during the development of the addiction, and a characteristic feature of a considerable part of addictive drugs i.e., dopamine-agonists properties [66]. The most important part of this system are dopaminergic neurons located in the VTA which project to the NAc and elaborate behavioral responses initiated by addictive drugs or other forms of rewarding stimuli [67]. Drug abuse changes the effectiveness of DA neurotransmission in synapses of the reward system structures, especially within NAc. The nature of these changes depends on a type of addictive substance [65,68]. The dopaminergic system has a multidirectional effect not only on the neuronal activity but also on the synaptic plasticity and molecular processes related to the gene expression and epigenetic modifications [69,70].

#### 1.4.2. Glutamatergic System

Glutamatergic projections terminating in NAc originate from Hip, Sub, Amg, thalamus (Th), VTA, and from the mPFC and prelimbic cortical areas [22,30,71,72,73,74]. The role of Glu in the BRS is associated not only with locomotor [75] and reward- or drug-seeking behaviors [76,77] but also with response-reinforcement learning [78].

Action of Glu on NAc is associated mainly with the development of LTP [79,80]. However, in particular cases, this process requires cooperation with other neurotransmitters, such as DA [59,63,64] or NE [81]. Involvement of several neurotransmitter systems is required for the effective functioning of the dendritic spines of MSNs in NAc [62,79]. Both AMPA and NMDA ionotropic glutamatergic receptors (iGluRs), as well as some metabotropic glutamatergic receptors (mGluRs) from group I (mGluR1 and mGluR5), are involved in a long-term potentiation (LTP) [72,80,82]. The unique role of AMPA receptors (AMPARs) in the NAc’s MSNs activation and LTP has been emphasized by some authors [79,83]. This is due to changes in their number occurring through externalization at extra-synaptic sites and trafficking into synapses, which significantly affects the synaptic strength [84,85,86]. The action of glutamatergic projection via AMPARs was demonstrated in both rapid and prolonged homeostatic plasticity [87]. It also requires a modulatory effect of DA, which affects insertion of the activity-dependent synaptic receptors [59] and synaptic scaling [60].

Activation of different types of glutamatergic receptors in NAc induces the development of dendritic spines in the MSNs. During maturation, they go through the stage of “silent” synapses with NMDARs, but not AMPARs [88]. Later on, they transform into “unsilenced synapses” characterized by the presence of AMPARs [42,89,90]. Glu plays an important role in the relapse mechanism during protracted drug use and compulsive drug seeking [91]. This can be explained by changes in the expression of AMPARs in the synaptic membrane [92,93], which results in enhanced effectiveness of Glu interactions within NAc [94,95]. Taken together, the presented data indicate a significant role of glutamatergic plasticity impairment within NAc in the disturbances of the goal-directed and motivated behaviors. A drug-induced long-term disruption of the balanced glutamate transmission leads to the addiction-related relapse vulnerability and enhancement of drug-seeking behaviors [41].

As mentioned before, the mechanism of drug addiction is associated with the modification of iGluRs (e.g., AMPARs and NMDARs), which is based on changes in their number and function [89]. First-time alcohol consumption has been shown to increase synaptic expression of the AMPAR GluA1 subunit and Homer proteins in NAc [96]. It also triggers plasticity processes in the D1R-containing synapses through enhancing mTORC1-dependent translation of proteins responsible for the stimulatory effect. On the other hand, prolonged ethanol withdrawal caused a reduction of the NMDARs expression, followed by the inhibitory effect in the hypersensitive mice [97]. Changes in the expression of AMPARs receptors in NAc are also responsible for cocaine exposure-induced affection of synaptic transmission and plasticity [79].

Altogether, the role of glutamatergic projection in the functioning of NAc involves various types of receptors, although the contribution of AMPARs and NMDARs is probably the most significant. Changes in the expression of these receptors correlate with morphological modifications of the spino-dendritic system, contributing to the development of synaptic plasticity and playing an important role in the processes of both short- and long-term synaptic potentiation.

#### 1.4.3. GABA-ergic System

The main target of GABA-ergic projection from NAc is pallidum (predominantly VP) [10,32,98]. Other functionally important GABA-ergic projections from NAc terminate in areas of BF responsible for acetylcholine (ACh) production. In these areas, GABA has a modulating effect upon ACh release, which is of importance for the appropriate functioning of neocortex and limbic structures [99,100]. Apart from these, other GABA-ergic projections from NAc terminate in several cortical, subcortical, diencephalic, and brain stem structures related to various functions. The characteristic distribution of the GABA-ergic efferent fibers coming from NAc suggests the role of this brain region in the coordination of the motor functions with some behavioral reactions based on emotions, mood, concentration of attention, and context-dependent arousal. Interestingly, the effects exerted by GABA in various brain areas are most likely to depend on its concentration. While in low concentrations it evokes hyperactivity, the effect can be the opposite when GABA concentration increases [10,101,102]. This mechanism provides the GABA-ergic system with the additional opportunity to influence the processes controlled by NAc.

The role of GABA-ergic projection and relevant receptors, necessary for the functioning of NAc and the whole BRS, have been raised in several excellent publications [2,42]. Among the most important functions of the GABA-ergic receptors are the following: regulation of release of the other neurotransmitters, alleviating the effects of stress and emotions, control of motor and metabolic functions, and modulation of the effects exerted by alcohol and addictive substances. Regulation of DA release in NAc is a complex process that involves GABAA and GABAB receptors (GABAARs and GABABRs, respectively) [103,104]. Both are located in the accumbal dopaminergic nerve endings and inhibit DA release. Activation of GABAARs in NAc induces disinhibition of local GABA signaling. This augments GABA release and, through interaction with GABABR, decreases DA efflux. Interestingly, inhibition of DA release due to activation of GABABRs leads to a decreased activation of delta1- and delta2-opioid receptors in accumbal GABA-ergic interneurons, whereas their stimulation has the opposite effect and increases DA efflux [105].

Some evidence suggests the presence of functionally diverse GABA pools in the axonal endings of neurons in NAc [106]. Newly synthesized GABA acts through the interaction with GABABRs. Apart from that, there is a certain amount of previously stored neurotransmitter that can inhibit the release of DA via the GABAARs, regardless of glutamic acid decarboxylase (GAD) inhibition which is responsible for the current GABA synthesis. Besides inhibition of DA release, GABA also exerts an inhibitory effect upon acetyl-cholinergic interneurons in NAc, via its interaction with GABAARs and GABABRs [107]. Stimulation of these receptors in cholinergic neurons in NAc results in a reduction of acetylcholine efflux [108]. Considering that the DA release in NAc from the axonal terminals of the VTA neurons is under the simultaneous control of the GABA-ergic (release inhibition) and cholinergic (release stimulation) systems, an appropriate level of activation of the cholinergic interneurons is required for DA release in NAc to balance the inhibitory effect of the GABA-ergic system and maintain the DA concentration at a level sufficient for the physiological functions of NAc [109]. Moreover, maintaining the balance between the cholinergic and GABA-ergic systems in NAc is important for the function of this structure in the striato-thalamo-cortical circuit [110]. Through this connection, NAc may influence the level of activity of the prefrontal cortex, by modifying the intensity of inhibition in VP and changes in the stimulation of the thalamo-cortical projection. Dysregulation of this system is responsible for the prefrontal cortex hyperactivity in the course of the obsessive-compulsive disorder [91].

The GABA-ergic receptors play an important role in controlling Glu release in NAc [111]. The parvalbumin-expressing GABA-ergic interneurons, interposed within the NAc microcircuits, stimulate GABAB heteroreceptors in glutamatergic terminals. Activation of these pre-synaptically expressed heteroreceptors causes a reduction in Glu efflux and, consequently, Glu-dependent synaptic efficacy in the D1- and D2-containing accumbal MSNs [111]. Consequently, by changing glutamatergic stimulation, GABAB heteroreceptors exert a significant effect on reward circuitry, selectively modulating glutamatergic transmission and NAc impact exerted upon other brain regions.

GABARs also play a significant role in the regulation of mechanisms related to the consequences of alcohol consumption. The reinforcing effect of ethanol in the NAc’s shell can be modulated by activation of GABAARs and GABABRs, together with 5-HT3 receptors [112]. Interestingly, the results of animal studies show that the effect of ethanol on the BRS, regulated by GABA-ergic receptors, is age-dependent [113]. Through the inhibitory effect exerted upon glutamatergic projections in NAc, GABAARs and GABABRs participate in enhancement of the inhibitory effect of ethanol, which leads to disruption in the reward system’s functioning. This effect has been more pronounced in adolescent than in adult mice [113]. This mechanism could explain the stronger and more uncomfortable feeling after alcohol drinking in adolescents than in adults.

Furthermore, GABARs play a crucial role in the regulation of addiction processes induced by psychostimulant drugs. For example, activation of GABAARs, but not GABABRs, modulates the reinforcing effects of morphine in NAc [114]. The GABABRs activation has also been shown to modulate behavioral and molecular processes related to reward feeling induced by nicotine consumption [115].

Additionally, the GABA-ergic system in NAc is involved in alleviating the effects of stress and emotions. Activation of the GABABRs in NAc ameliorates spatial memory impairment after stress exposition [116], and exerts an anxiolytic effect in the rat model of stress [117]. Another function of GABAARs in NAc, together with the D1R and D2R, is modulation of motor activity, which also involves extensive reciprocal connections and cooperation with other basal ganglia nuclei [118]. Finally, stimulation of GABAARs and GABABRs in the NAc shell increases feeding in satiated rats [117,119]. This suggests another, although so far poorly explored, physiological function of the shell related to the GABA-ergic control of vegetative functions.

In summary, GABA-ergic receptors in NAc have important regulatory functions associated with direct inhibition of other cell populations and controlling the release of several neurotransmitters. Regulation of NAc activity via GABA-ergic receptors enables precise control of processes occurring in the BRS.

#### 1.4.4. Serotoninergic System

Large projections originating from the small population of neurons concentrated in the raphe nuclei complex (e.g., DRn) deliver 5-HT to the BRS. This monoamine neurotransmitter is involved essentially in all physiological processes controlled by NAc. The 5-HT function in this brain area depends on the dynamic balance between other neurotransmitters’ systems and concentrations of the relevant neurotransmitters. One of the important functions of 5-HT in NAc is its role in motivation. This function is closely related to the facilitating activity of DA. Impairment of interactions between the serotoninergic and dopaminergic systems may result in anhedonia, lack of motivation, and finally in depression [120]. Interestingly, 5-HT has different actions upon various structures of the BRS. While its effect upon NAc is generally related to the enhancement of motivation, its impact on VTA is inhibitory [121].

Another important function of 5-HT in NAc is the regulation of prosocial interactions and behaviors, which is closely related to the rewarding effect of such interactions [122]. Additionally, results of animal studies have shown that an interaction between 5-HT and oxytocin is required as signal reinforcement for normal social relations [123]. Consequently, 5-HT deficits may lead to disruption of social relations and contribute to the development of some psychiatric disorders (e.g., autism).

The role of 5-HT in NAc is also associated with the reinforcing effect of ethanol. This effect is further enhanced by DA release, which leads to an increased reward experience and impulsivity. In combination with some drugs, such as mephedrone, 5-HT and DA can increase the susceptibility to alcohol abuse, due to their increased release in NAc and mPFC [124]. The increase in 5-HT, as well as DA and NE content in NAc might also occur after administration of cocaine [125] and monoamine uptake blockers [61]. However, there are some premises indicating that, apart from contributing to the addictive effect, 5-HT may also participate in reducing the risk of addiction. Serotonin can exert such an effect when administered along with some hallucinogenic agents from the group of indoleamines and phenylalkylamines (e.g., psilocybin and lysergic acid diethylamide) [126].

The serotonergic system has a complex influence on NAc. This is due to the large number of receptors, their distribution on many types of cells, and to the use of several neurotransmitters. Among different types of receptors, at least two representing the 5-HT2R group (i.e., 5-HT2AR and 5-HT2CR) play an important role in control of addiction mechanisms. Taking into account the fact that development of addiction is associated with an increase in DA released in the NAc, it has been suggested that the role of these receptors is based on a regulation of the DA concentration [127]. They can either intensify or weaken the effects of psychoactive and addictive drugs, and thus indirectly affect behavioral reactions [126]. There is evidence for the opposite effects of stimulation of 5-HT2A and 5-HT2C receptors leading in the first case to an increased DA release, and in the second to its decrease [128,129,130]. On the one hand, activation of the 5-HT2AR may initiate or augment drug craving and relapse behaviors [126]. On the other hand, 5-HT2AR antagonists can inhibit drug-seeking or drug-taking behaviors [131]. This explains attenuation of the stimulatory effects of cocaine and amphetamine [132,133,134]. It has been shown that 5-HT2CR stimulation inhibits self-administration and the addictive effects of drugs [135,136,137]. The 5-HT action at this receptor is believed to be the primary mechanism responsible for its anti-addictive effect upon the NAc. Selective agonists of this receptor have a similar effect [126]. The molecular mechanism is based on activation of the 5-HT2CR in GABA-ergic neurons in the VTA, which inhibit stimulation of the dopaminergic neurons, thus reducing the release of DA from their axonal terminals in the NAc [121,136,138,139]. In addition, 5-HT2CR also modulates the DA signaling at the postsynaptic level in the NAc core [140]. Hence, two types of serotonergic receptors 5-HT2AR and the 5-HT2CR allow the 5-HT acting upon the NAc to influence the development, as well as alleviation or inhibition, of the addiction process [126,128]. Furthermore, it is believed that selective antagonists and agonists acting on 5-HT2AR and 5-HT2CR, respectively, may contribute to alleviation of addiction [126]. The role of other serotonergic receptors, such as 5-HT2BR, in the regulation of drug addiction process is still not fully elucidated [141,142,143].

Apart from the aforementioned agonists and antagonists of the serotonergic receptors, other drugs such as partial agonists of serotonergic receptors, inhibitors of the 5-HT transporters and multiple neurotransmitter uptake inhibitors also act on the serotonergic system [144,145]. Their contribution relies on changes in the 5-HT concentration, prolongation of its action, inhibition of reuptake, different specificity and selectivity [146,147]. In addition, their action is associated with different affinity to receptors located in various types of neurons, using several different neurotransmitters, as well as with the interaction between neurotransmitter systems [61,148]. All this explains the variation in the efficiency of drugs and initiation of different behavioral response patterns after their use. It is worth noting that, whereas the experimental studies on candidate antidepressant substances yielded promising results, development of drugs having sufficient specificity and effectiveness requires further research [126,128].

In addition to all the above-mentioned functions, 5-HT also plays a regulatory role in the metabolic processes in NAc, leading to an increase in glucose blood levels [149]. Studies show, on the one hand, the significant role of 5-HT in the regulation of several processes controlled by NAc. On the other hand, they emphasize the importance of cooperation among all neurotransmitter systems in controlling these processes.

Although stimulation of different types of 5-HT receptors in NAc can enhance the effect of some addictive substances such as alcohol, stimulation of other types of receptor in this brain area leads, paradoxically, to a lower likelihood of addiction in response to the other psychostimulants [112,126]. The reinforcing effect of ethanol is based on 5-HT interaction with 5-HT3 receptor (5-HT3R), in cooperation with GABA-ergic system and activation of GABAARs and GABABRs in the NAc shell [112]. However, 5-HT may also play a different role. By acting on 5-HT2C receptors localized in the GABA-ergic MSNs in the NAc shell, it induces inhibition of the potassium Kv1.x channels activated by classic hallucinogens, such as indoleamines and phenylalkylamines [126]. The presented mechanism of 5-HT action could explain the lower likelihood of addiction induced by these substances.

#### 1.4.5. Noradrenergic System

NE, as one of the most important brain neurotransmitters, is also represented in the BRS. Its sources are neurons localized in TegB, mostly in LC. Extensive projections of this relatively small population of neurons allow the distribution of NE to almost all brain regions. Under stressful and rewarding stimuli, NE is released in NAc and mPFC [150]. The amount of released neurotransmitter depends largely on the nature of the stimulus. This emphasizes the importance of NE in the regulation of behavioral responses. Due to a wide range of noradrenergic projections, reaching not only NAc but also AmgBL and PFC, NE is an important factor regulating social interactions [151]. Functional balance between dopaminergic, serotoninergic, and noradrenergic systems in NAc is crucial for maintenance of the appropriate level of motivation and hedonia [120]. On the other hand, dysregulation of this balance can lead to depression. Additionally, NE plays an important role in the formation of fear memory, which results from its coordinated action exerted upon NAc, dHip, and mPFC [152]. Moreover, the results of animal studies have shown that NE administration into NAc induced an increase in 24-h water intake. This suggests another important function of NAc as a water balance and drinking behavior controlling center [153]. Finally, NE released in NAc can modulate pain sensation in morphine-dependent rats [154].

Another study has shown that cocaine administration enhances the release of NE, DA, and 5-HT in NAc [155]. The dynamics of this process are different for each neurotransmitter. Stimulated release of NE enhances DA efflux and its action on NAc [61]. Interestingly, the release of NE in NAc is controlled by DA and its effect may be either stimulatory or inhibitory, depending on DA concentration. At moderate DA concentration, NE release is inhibited mainly by D2 receptors in appropriate axonal terminals, whereas, at increased DA concentration, NE release is stimulated by D1 receptors [156].

In addition to the abovementioned functions, increasing evidence points to NE involvement in the mechanism of alcohol addiction. Karkhanis et al. reported that chronic early-life stress resulting from social isolation has an impact on the behavioral risk of alcoholism manifested by a greater tendency to alcohol self-administration [157]. This could be explained by an increased sensitivity to NE and DA, as well as an increased NE and DA release in NAc in response to alcohol administration. This interesting mechanism indicates an important role of functional interrelationship between the main neurotransmitter systems in NAc and addiction.

Altogether, NE via its action in NAc, as well as in other BRS structures, regulates a wide spectrum of physiological processes and plays a role in the development of addiction. Moreover, cooperation with other neurotransmitters, such as DA or 5-HT, determines the NE action upon NAc and its regulatory role in these processes.

NE plays a role in several processes, such as concentration of attention, wakefulness, drug addiction, and psychostimulants relapse. Action of this neurotransmitter within NAc is based mainly on its interaction with the α1-adrenergic receptor (α1AR) [158]. A rare colocalization of α1bAR with D1R has been reported in postsynaptic elements of neurons within the shell of NAc [158]. The characteristic distribution pattern of α1bARs (mostly in unmyelinated axons and axon terminals, and less often in dendrites), suggests that the function of these receptors (and consequently of the whole noradrenergic system) is based on the regulation of activity of other neurons and synchronization of the release of the other neurotransmitters within the NAc. Another type of adrenergic receptor identified in NAc is the α2-adrenergic receptor (α2AR), whose function is related to the reduction of NE release, but not DA release, in this brain area [159]. The effect of α2AR activation on DA release in NAc is indirect. When activated with NE, α2ARs present in dopaminergic neurons in VTA cause reduced DA release via the axonal terminals localized in NAc [159]. Thus, the action of NE in NAc via specific receptors is based mainly on regulation of the release of other neurotransmitters and modulation of the activity level of the projection neurons.

In summary, the release of neurotransmitters in NAc is precisely adjusted to the cause of activation, the goal to be achieved, and the origin of the activated projection. The action of a neurotransmitter depends on its concentration in NAc and its binding to specific receptors. The important role of neurotransmitter receptors the NAc functioning is related to changes in their expression level, subunit composition, and externalization or displacement within the plasma membrane or outside the synapses. The cooperative effect and synergistic action of receptors representing different neurotransmitter systems are critical for motivation, learning, and addiction.

## 2. Neuroglia Participates in a Wide Spectrum of Physiological and Pathological Processes within the Nucleus Accumbens

**Figure 4:**
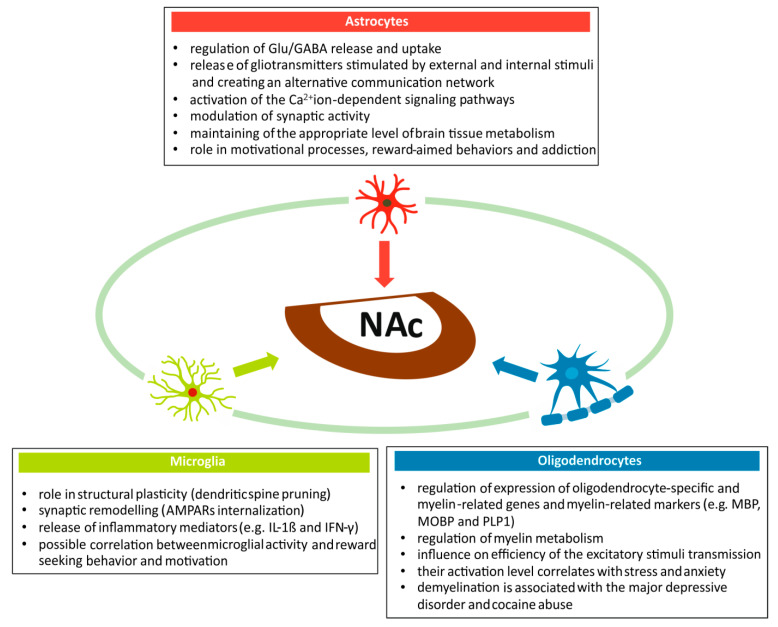
**Neuroglia function in nucleus accumbens**. Neuroglial cells play an important function in the regulation of not only physiological but also pathological processes in NAc. The role of astrocytes is maintenance of the signaling pathways through providing NAc with neurotransmitters, modulation of synaptic activity, and establishing an alternative communication network due to secreted gliotransmitters. In addition, astrocytes are responsible for maintaining an appropriate level of brain tissue metabolism. Microglia, through release of the inflammatory mediators, influence morphological and functional neuroplasticity and modulate neuronal activity. Oligodendrocytes are involved in regulation of the expression of the myelin metabolism-related genes. Consequently, through maintaining the transmission efficiency of excitatory stimuli along the nerve fibers, these cells have the potential to influence activity and plasticity processes in the BRS. All neuroglial cell subpopulations play an important role in the pathological processes affecting the NAc integrity, although their contribution to these processes is still poorly understood.

The proper functioning of the BRS requires cooperation among all morphological elements of brain tissue. Structural and functional relationships occurring between neuroglia and neurons form the basis not only for processes of motivation and reward-aimed behaviors, but also for the development of addiction and mental diseases (Figure 4). As can be inferred from the results of previously published studies, the role of neuroglial subpopulations in the NAc function could be significant, although it is still poorly understood. This warrants further research in the field.

Astrocytes are an important element of the brain tissue involved, together with neurons, in the regulation of reward and addiction mechanisms [160]. An important function of astrocytes in NAc is participation in Glu/GABA release and uptake, and activation of the Ca^2+^ ion-dependent signaling pathways [161,162]. As a component of the tripartite synapse, astrocytes influence synaptic activity in NAc and, by releasing gliotransmitters and neuromodulators, they modulate the response generated by external stimuli influencing motivation, reward-aimed behaviors, and addiction [163]. Finally, resistant to fluctuations in cerebral blood flow, astrocytes are responsible for maintaining an adequate level of brain tissue metabolism in physiological and pathological conditions.

Interesting data about the role of microglia in NAc come from animal studies. Microglia activation has been reported in mice fed with a high-caloric chocolate cafeteria diet [164]. Apart from weight gain, this resulted in a modification of structural plasticity represented by dendritic spine pruning (removal of synapses) and synaptic remodeling, initiated by microglial release of inflammatory mediators, such as interleukin-1β (IL-1β) and interferon gamma (IFN-γ). The effect of mediators released by the activated microglia in NAc on the dendritic system and spines depends on external stimuli acting on the BRS. Thus, a decrease in dendritic spine density in the shell corresponds to decrease in the sense of reward in animals fed *ad libitum*. On the other hand, an increase in the dendritic spine density correlates with compulsive seeking behavior [165]. In cases of drug abuse, an activation of microglia has been reported. Repeated cocaine administration triggered microglia activation in Str, leading to increase in tumor necrosis factor α (TNF-α) levels and internalization of synaptic AMPA receptors [166]. This resulted in inhibition of synaptic plasticity and behavioral sensitization.

Oligodendrocytes, like other neuroglial cell subpopulations, play an important role in the BRS, both in physiological conditions and pathology. Their role in myelin metabolism is important for the functioning of the brain tissue. Through controlling myelin metabolism, oligodendrocytes can influence the plasticity processes related to transmission efficiency of excitatory stimuli along the neuronal fibers. Consequently, myelin metabolism seems to be a good indicator of the BRS status during stress or anxiety. Down-regulation of myelin genes and oligodendrocyte-specific genes in NAc and PFC was recorded after four weeks of stress exposure in mice [167]. Similarly, chronic social defeat stress initiated region-specific differences in myelination. After exposure to this type of stress, decrease in myelin protein content was observed in the limbic areas, including NAc [168].

Another important issue represents myelination disorders and changes in myelin synthesis, resulting in impairment of the brain function observed in the course of some mental illnesses. The major depressive disorder (MDD) is associated with changes in the myelin content in several brain regions, but in particular in structures of the limbic system, including NAc [169]. Further research is needed to explain the relationship between the severity of emotional, cognitive, and behavioral symptoms and extent of changes in myelin content within the limbic system.

Results of a postmortem study showed myelination impairment in several brain areas, including NAc, after chronic cocaine abuse [170]. Dysregulation of myelin metabolism results from alterations in gene expression. A reduced expression of proteins encoded by myelin-related genes, such as myelin basic protein (MBP), myelin-associated oligodendrocyte basic protein (MOBP), and proteolipid protein 1 (PLP1) was observed after cocaine administration [170]. Because PLP1 is crucial for myelin stability, a decrease in expression of this protein can be an indicator of changes in myelin structure in consequence of chronic cocaine abuse. Furthermore, reduction in the number of MBP-immunoreactive oligodendrocytes was observed in the NAc after cocaine intake [171]. Other interesting data showed inhibition of white matter loss in NAc during cocaine withdrawal after chronic abuse in mice treated with ceftriaxone [172]. Nonetheless, the mechanism of action and potential strategies for therapeutic application of this antibiotic in addiction therapy require further research.

To conclude, neuroglia participates in a wide spectrum of processes occurring in NAc and other areas of the BRS. Its role includes modulation of synaptic transmission and signaling pathways within tripartite synapses, as well as regulation of energetic metabolism by astrocytes. It also takes part in the regulation of the BRS activity, through the release of inflammatory mediators by activated microglia. Finally, these cell populations regulate myelin metabolism, expression of oligodendrocyte-specific proteins and, consequently, the efficiency of stimuli conduction along the neuronal fibers. The modifying effect of neuroglia upon the BRS activity (including NAc) involves both physiological processes and a wide spectrum of pathologies associated with addiction, neurodegeneration, and mental illnesses. Further studies on the role of neuroglia in these processes are needed.

## 3. Nucleus Accumbens Is Responsible for Executive Behaviors Aimed at Motivation, Survival, and Reward Achievement

In the 1980s, Mogenson and colleagues formulated a hypothesis that NAc functions as an interface between the limbic and motor systems [10]. More recent studies have confirmed this concept, extending it with new morphological and functional data [1,3,42,47,173]. Due to its localization on the border between the limbic and motor systems, and its extensive connections, NAc can integrate stimuli coming from different brain areas. For example, it coordinates emotional inputs originating in Amg with stimuli enhancing motivational drive, resulting from the interaction of dopaminergic and serotoninergic signals generated in the brain stem and diencephalic areas. Moreover, NAc receives contextual information from Hip, and information about the current level of attention from MThn and IThn. This integrating function of NAc is then precisely coordinated with cognition, planning, and execution processes developed in the PFC. Hence, the role of NAc is to integrate executive behavior, motor reactions, motivation, learning and memory, and vegetative reactions important in physiological conditions. All these processes are important for both an individual’s survival and survival of the species [174,175]. These activities could be manifested in feeding [176], sexual [177], and risk-undertaking behaviors, which are aimed at reward achievement and pleasure [178]. NAc has also been involved in learning processes. Results of animal studies showed an important role of this BRS area in place preference behaviors [179,180], and in the avoidance of life-threatening situations [181]. Other studies reported that NAc modulated incentives to achieve rewards of both a natural and unpredictable character [182,183]. Finally, NAc is involved in drug addiction [184].

Differences in functioning of the two parts of NAc have previously been reported [42,50,185,186,187]. The shell, activated by external stimuli or substances, strengthens the reward feeling. This part of NAc also plays an important role in shaping innate and unconditioned behaviors related to feeding and defense. This is related to biological drives based on cooperation among visceral, limbic, and motor systems [3,42,187]. Additionally, the medial part of the shell is believed to be involved in strengthening of the novelty effect [183,188] associated, for example, with feeding behavior [189], but also with the administration of substances having rewarding properties, including psycho-stimulating drugs [190,191,192].

The core of NAc is involved in responses to motivational stimuli [193], impulsive and emotional responses [194,195], responses developed during instrumental learning and, finally, conditioned responses [188,196]. There is also evidence of the core involvement in the spatial learning processes [197]. Although most of the studies connect the function of NAc to positive emotional responses, some studies suggest its role in aversive motivation [198] and, together with Amg, in the elaboration of negative emotional responses [199].

## 4. Stress, Psychostimulants and Experience Impact NAc Function during Early Development and Adolescence

Like other brain regions, NAc undergoes characteristic morphological and functional changes during ontogenesis. At the subsequent stages of development, changes in the cellular structure, formation of connections with other brain areas, along with development of neurotransmitter systems and signaling pathways, as well as development of structural and functional plasticity, occur [200,201]. Numerous reports suggest that the effects of such factors as stress, drugs and various forms of addiction, as well as experience during development, are different from the effects of these factors in the adult period [202,203]. Importantly, although it is still poorly understood, the action of these factors affects the further development and functioning of NAc in adulthood.

The action of the above-mentioned factors at different stages of ontogenesis results in development of behavioral disorders and mental dysfunctions either during adolescence or in adulthood. This could be a consequence of an impairment in functioning of the endocrine system (e.g., hypothalamus-pituitary-adrenal axis; HPA), disturbances in the functioning of neurotransmitter systems (e.g., dopaminergic, serotonergic, glutamatergic, GABA-ergic, noradrenergic and others), as well as changes in the expression of neurotrophic factors (e.g., BDNF) and transcription factors (e.g., pCREB, deltaFosB) [204,205,206,207,208].

### 4.1. Mechanisms and Effects of Stress Acting on Nucleus Accumbens during Early Development and Adolescence

During early development, due to an incomplete development of endocrine regulatory mechanisms, stress hormones (glucocorticoids) may be harmful to immature NAc, and induce abnormal behavioral responses [209,210]. Defense mechanisms during that period are based on a higher threshold of the HPA activation and attenuation of the stress response [211]. The result of these processes is the stress hypo-responsive period [211,212]. This is characterized by a rise in the threshold of excitability of the HPA axis, and activation only after the action of very strong stimuli [213]. The long-term effect of mild stress during this period may, by increasing the level of glucocorticoids, lead to a decrease in a production of BDNF [204,214]. A protracted consequence of this process may be a disturbance of the structural and functional plasticity in NAc, which is manifested by disturbances in the formation of the spino-dendritic system and even the death of neurons [215]. The negative consequences of these phenomena are present not only during development, but also in adulthood. In the case of NAc, they may manifest themselves as behavioral responses to environmental stimuli such as stress and addictive substances.

Adolescence is the period in NAc development characterized by the final formation of connections, shaping balanced functional relations between neurotransmitter systems, as well as stabilization in the production level of neurotrophic factors and balanced gene expression. In addition, the development of hormonal maturity related to the HPA axis, both in terms of controlling stress reactions and achieved sexual maturity, is important for the functioning of the reward system during that period. Despite these changes, during adolescence there is a greater susceptibility to stressful and aversive stimuli than in adulthood [214]. This can result in functional disorders manifested by many symptoms, such as anxiety, aggression or depression, having either a transient nature or persisting later in life [216]. It should be noted, however, that the relationship between the effects of stress in adolescence and occurrence of the psychopathological disorders in adulthood is complex and requires further research.

### 4.2. Mechanisms and Effects of Nucleus Accumbens Exposition to Addictive Substances during Development

The susceptibility of NAc to the harmful effects of addictive substances during development can be illustrated by the effects exerted by nicotine. Exposure to this commonly consumed psychostimulant has been linked to NAc impairment. The most important research includes the effects of early exposure to nicotine and its long-term consequences, the effects of nicotine withdrawal during development and adolescence, and mechanisms shaping the reward feeling triggered by nicotine during development. It has been shown that exposure to nicotine already in the fetal period affects the expression of genes of growth factors, death receptors, and some kinases related to the regulation of cell death or survival in adolescence [217]. Maternal smoking induces a modification of cell adhesion molecules (CAM) such as neurexin, immunoglobulin, cadherin, and adhesion-GPCR superfamilies in the fetus [218]. The CAM-initiated signal transduction is modified by a gestational nicotine treatment. In the NAc, it may reduce a number of the excitatory synapses which can lead to neurobehavioral deficits in adolescence. Furthermore, exposure to nicotine during adolescence, which is a time window for sensitivity to its effects, reduces cognitive abilities and diminishes attentional performance in pursuit of the goal of satisfaction and reward in adulthood [219].

Nicotine withdrawal has different effects on the functioning of NAc in adolescence as compared to adulthood. Studies have shown that discontinuation of nicotine in juveniles causes less side effects than in adults [220]. This may be due to an underdevelopment of the GABA-ergic system and a weaker inhibition of dopaminergic neurons in the VTA, which, to a lesser extent, reduces the dopamine content in NAc. Negative aversive symptoms resulting from nicotine withdrawal are less pronounced in adolescence [221]. It is believed that the glutamatergic system supporting DA release and action in NAc is more developed than that of the GABA-ergic system. As a result, in young people, nicotine increases the reward effect, weakening negative reactions. Thus, the development of neurotransmitter systems and plasticity in NAc influence the nature of the interaction of the nicotine-stimulated dopaminergic projection.

### 4.3. Reward Mechanisms in Adolescence—Role of the Neurotransmitters and Neurotrophic Factors

There are premises indicating involvement of different mechanisms triggering the reward and satisfaction feelings at various ages [222]. These can be triggered by different neurotransmitters depending on the stage of development. Apart from DA, other neurotransmitters such as NE and 5-HT, whose concentration is increased in NAc, could be involved in these processes [222]. Greater nicotine preference and its effect in juveniles may depend on the content of neurotransmitters and on a different composition of receptors in the NAc shell, as well as on changes in neuropeptide expression, compared to adults [223]. An exposure to nicotine during adolescence initiates structural plasticity changes such as an increase in the number and length of dendrites [224]. Furthermore, peri- and post-adolescent nicotine exposure induces an increase in FosB expression in NAc and the hippocampus [225]. This, in turn, contributes to the increase in the activity of these structures related to the sense of reward and memory.

### 4.4. Influence of Learning and Gained Experience on the NAc Function in Adolescence

Adolescence is a period of development associated with intensive learning, gaining various types of experience, as well as developing a responsiveness to stressful stimuli resulting from action of environmental factors and social interactions [226]. All of these significantly affect the functioning of the BRS, including NAc. It should be noted that the behavioral reactions developed and shaped during this period may differ significantly from those occurring in adulthood. Adolescence is a period associated with gaining experience in social relations, characterized by the choice of behaviors and decision-making frequently involving risk of uncertain consequences [226]. The neurobiological basis of such behaviors is related to incompletely shaped interactions among the three functional brain systems: the reward system (with a significant role of NAc), the limbic system related to emotional activity (represented by the amygdala), and the coordinating system (with contribution of the ventro-medial prefrontal cortex) [226]. The disproportion in the development of these three systems, with the predominance of NAc and amygdala development over the prefrontal cortex, may explain the specificity of behavior and decision choices in adolescence, as well as the susceptibility to psychopathological disorders, including depression and anxiety.

Chronic juvenile (pre- and adolescent) stress of different nature has a significant impact on development of many brain areas, including NAc and the prefrontal cortex, important for shaping of the reward processing and the executive functions. Disturbances in the functioning of these areas resulting from impact of stressful stimuli on the immature structures may manifest in psychopathological symptoms during development and in adulthood, and sometimes may lead to the development of mental illnesses [227].

Some evidence indicates that early-life adversity experiences like poverty, chaotic environment, maternal separation or poor parental care may significantly contribute to dysfunction of the BRS [228]. They may lie at the base of the affective disorders, such as depression and anhedonia at the later stages of development. Moreover, they also make those in that stage prone to the development of addiction. One of the patho-mechanisms at the base of these disorders is associated with changes in the expression of the corticotropin-releasing factor (CRF) and, indirectly, with decreased effectiveness of the connections between NAc and amygdala nuclei involved in fear and anxiety reactions, with a simultaneous impairment of the pleasure and reward reaction [228].

## 5. NAc Participates in Elaboration of Aversive Reactions

The plasticity processes occurring during adolescence in the shell and core of NAc not only have different dynamics but also reveal a different involvement in rewarding and aversion effects, thus emphasizing the functional differentiation of both parts of this nucleus [229]. Some authors have suggested that the functional differentiation between the NAc shell and core is due to involvement of the D1 and D2 receptors in the reward and aversion reactions [230]. Stimulation of the D1 receptors in the medium spiny neurons (D1-MSNs) in the NAc leads to generation of the reward-related response, while activation of the D2 receptors in MSNs (D2-MSNs) is responsible for aversion [230]. Another concept states that both the D1- and D2- receptors containing MSNs control reward and aversion, and the nature of the reaction generated in NAc is determined by the pattern of neuronal stimulation [231]. While brief optogenetic stimulation of D1- or D2-MSNs elicited a positive reinforcement, their prolonged activation induced an aversion. Moreover, the final type of response is also influenced by the activity status of the opioid system [231]. Blocking κ-opioid receptors in the VTA eliminates results of the D1-MSN prolonged stimulation, whereas blockade of δ-opioid receptors inhibits behavioral response initiated by the D2-MSN prolonged stimulation. According to other authors, the glutamatergic projection originating from neurons located in the VTA and reaching NAc may play a significant role in the formation of the aversive reaction. By stimulating AMPA receptors in asymmetric synapses on parvalbumin-containing GABA-ergic interneurons leading to their activation, output MSNs of NAc are inhibited, which ultimately results in the aversive reaction [232]. Finally, other reports show functional differentiation between the NAc regions associated with different distribution of dynorphinergic cells involved in generation of the aversive and rewarding reactions [233]. The photo-stimulation of the dynorphin cells localized within the ventral part of the NAc shell is responsible for the formation of the aversive behavior, while stimulation of dynorphin cells in the dorsal part of the NAc shell favors positive reinforcement in the place preference test in mice [233]. Finally, some studies suggest that dynorphin induces negative affective symptoms related to nicotine withdrawal, such as anxiety, aversion and decrease of reward system function [234,235].

Overall, the presented data indicate the complex character of the NAc function in the rewarding or aversive responses. This implicates the existence of different regulatory mechanisms depending on the situational context of the shaped reaction, which are disturbed in the course of such pathological processes as addiction, stress, depression or mental disorders.

## 6. Mechanisms of Neuroplasticity within Nucleus Accumbens

### 6.1. Morphological Changes in the Dendritic Tree and Dendritic Spines of the Accumbal Neurons Are Triggered by Both External and Internal Stimuli

The importance of dendritic spines in the NAc neurons results not only from their involvement in synaptic transmission and plasticity, shaping motivational behavior and reward feeling, but also from their role in the development of addiction. Morphology of dendritic spines in NAc, predominantly bearing excitatory synapses, is determined by processes that occur both in the prenatal and postnatal period [236]. The development of the dendritic system is influenced by factors such as growth regulators, stress, learning, administered drugs and psychostimulants. All these factors influence the shape and density of dendritic spines, which develop according to different patterns in various brain areas, such as NAc, PFC and Hip [237,238]. Maturation of dendritic spines in NAc is based on morphological changes from the stage of thin spines to mature mushroom-shaped ones [42]. Learning and memory processes improve the efficiency of synaptic plasticity, also through changing the morphology of dendritic spines in Hip neurons [239,240]. While thin spines are associated with learning processes, mature mushroom-shaped spines are related to long-term memory and the maintenance of neuronal networks in Hip [241]. In addition, mushroom-shaped spines with small heads are characteristic for weak or silent synaptic connections [242]. Long-term stress affects the morphology and density of dendrites and dendritic spines in NAc, promoting their atrophy and decrease in number [236]. This results in spine reduction in some brain areas, such as Hip and PFC, but also in an increase in others, such as Amg and NAc. These chronic stress-induced changes occur via activation of signaling pathways involving cAMP-ERK1/2-CREB, TNFα-NF-κB, and Ras-ERK [243,244,245].

Drugs of abuse, such as cocaine, change the number and structure of dendritic spines in NAc [43,44,246]. Cocaine-induced reward and seeking behaviors are driven by morphological, neuroplastic and functional changes in NAc [41,247,248,249,250]. An important factor responsible for the cocaine-induced changes in the dendritic spine morphology is a small GTPase, Rap1b [42]. The increase in the expression of this protein occurs after cocaine exposure and correlates with the morphological changes of dendritic spines in the NAC neurons, which initially show an increase in the number of immature spines with a concomitant decrease in synaptic strength [251]. Later on, an increase in both mature spine density and in synaptic strength are observed. These morphological changes in NAc correspond to behavioral reactions based on an increase in locomotor activity directed at cocaine searching and accompanying increase in reward feeling. Similarly, activation of the BDNF-tyrosine kinase B (TrkB) signaling pathway, with the following activation of pERK-dependent cascades, has been shown to induce spine formation in the hippocampal neurons [252,253].

In summary, both external and internal stimuli can initiate morphological changes in dendritic spines in NAc and other regions of the BRS. The importance of dendritic spine modifications results from their involvement in the synaptic transmission and neuroplasticity processes. These modifications are accompanied by activation of signaling pathways contributing to an increase in efficiency of synaptic transmission, which enables specific behavioral reactions. The modifications occur not only in physiological processes, but also in pathological processes, such as addiction, mental, and neurodegenerative diseases.

### 6.2. Mechanisms of Synaptic Neuroplasticity within the Spino-Dendritic System of the Reward-Related Brain Areas Require Changes in Gene Expression and Activation of Specific Signaling Pathways

Growing evidence shows the relationship between stress, taken psychostimulants, and some mental disorders and changes in the synapto-dendritic system of the reward-involved brain structures [42,44,89,95]. However, the molecular bases of these relations remain poorly understood. An example of such a relationship are changes in the activation of genes encoding cytoskeleton regulatory proteins, observed in stress and depression [254]. Remodeling of the actin cytoskeleton in the BRS in response to addictive substances exposure has been extensively demonstrated [255,256,257]. Up-regulation of GTPase RhoA and stimulation of its effector Rho-kinase (ROCK) result in dysregulation of the production of actin, a protein which is one of the most important components of the cellular skeleton’s microfilaments [254]. This initiates the reconstruction of dendritic tree leading to a reduction in its complexity [258,259,260,261]. Loss of dendritic spines is followed by atrophy of the dendritic arbor, which finally results in the reduction of synaptic drive in the MSNs with dopaminergic D1 receptor (D1R-MSNs) in NAc.

Some addictive substances can also induce molecular changes in the BRS. Cocaine and morphine induce a decrease in content of Homer1 protein and postsynaptic density of protein 95 (PSD95) in the BRS [43,262]. The levels of small G proteins such as Rho, Rac1, and Cdc42 are also reduced after morphine consumption [262]. These proteins trigger signaling pathways which initiate remodeling of actin in the cellular cytoskeleton. All of these are activated by guanine nucleotide exchange factors (GEFs), which are responsible for the conversion of GDP into GTP. Further activation of the regulators of cytoskeleton transformation requires activation of GTP-ases, which occurs after their binding to GTP [43,262]. Therefore, changes in the spino-dendritic system caused by disturbances in production of cytoskeleton regulatory proteins are an important feature of the BRS dysfunction, manifested by plasticity disorders and development of addiction.

A recent study found a correlation between drug addiction and dysregulation of the circadian rhythm of sleep and wakefulness [263]. These disturbances can promote the appearance or intensify already present drug abuse in susceptible persons. The neuronal PAS domain 2 (NPAS2) protein, which regulates the circadian cycle, plays an important role in the regulation of glutamatergic neurotransmission in the MSNs of NAc [263]. NPAS2 also has a modulatory effect on synaptic plasticity, affecting the strength and sensitivity of the excitatory synapses in D1R-MSNs in NAc. In this way, it modulates the cocaine-induced reward-related behavior. As NPAS2 inhibits synaptic plasticity in NAc, a decrease in its concentration in the D1R-MSNs results in an increased cocaine preference. Results of preclinical studies have shown that downregulation of NPAS2 in D1R-MSNs in NAc modifies a cocaine-conditioned behavioral response and a cocaine-conditioned place preference in mice [263]. A selective influence on the NPAS2-containing subpopulation of D1R-MSNs in NAc could be, at least in theory, a good strategy allowing for precise control over the mechanisms of reward- and addiction-related processes in the subpopulation of projection neurons in NAc.

Further research on the molecular basis of plasticity mechanisms in the spino-dendritic system in the BRS is warranted, since it might contribute to a better understanding of the causes of numerous pathological processes and to the development of new and more effective therapies.

## 7. Role of Neurotransmitters Transporters and BDNF in Synaptic Plasticity within Nucleus Accumbens

### 7.1. Neurotransmitter Transporters

The main role of neurotransmitter transporters in the CNS is removal of the appropriate mediators from the synaptic cleft [264]. Therefore, they exert a significant effect on the strength and duration of stimulation in synapses belonging to various functional systems represented in a given brain area. By changing the affinity and the duration of action, they can indirectly influence the final effect induced by a specific stimulus. In the case of NAc, transporters of neurotransmitters such as DA, Glu, GABA, 5-HT and NE are of practical importance.

From a practical point of view, the effects of inhibition of the individual transporters are of great importance, on the one hand affecting the dynamics of psychopathological disorders such as depression, anxiety, obsessive-compulsive disorder, attention deficit hyperactivity disorder (ADHD), and addiction [264]. On the other hand, they are of interest in research focused on searching for effective treatments for these disorders.

### 7.2. DA Transporter (DAT)

DAT plays an important role in eliminating DA from the synaptic space, ensuring physiological homeostasis in the neurotransmitter system [265]. By inhibiting its action, it is possible to alleviate psychopathological symptoms in the course of diseases such as schizophrenia, ADHD, bipolar disorder, and Parkinson’s disease.

Bahi et al. reported the crucial role of DAT in ethanol-seeking behavior, as well as acquisition and retrieval of ethanol contextual memory in mice [266]. Consequently, by influencing DAT expression it is possible to modulate the rewarding properties of ethanol. Inhibition of DAT enhances DA neurotransmission induced by cocaine [267]. The addictive cocaine effect is mediated primarily by blocking DA uptake, while NE and 5-HT have modulatory roles [268]. Alterations in DA uptake inhibition are responsible for the rewarding and addictive properties of cocaine. Tolerance to cocaine’s effects is considered as an enhancing factor for this drug taking behavior [269]. The reinforcing effect of cocaine’s addictive properties is based on an action of the dopaminergic system in NAc through inhibition of DAT. There are alternative ways to achieve such an effect e.g., by modulation of the serotonergic system function in the VTA, which results in an increase of DA levels in NAc [270]. Interestingly, results of study by Siciliano et al. have shown that exposition to amphetamine can lead to decrease in cocaine intake by reducing cocaine tolerance [271]. It has been suggested that amphetamine contributes to the deconstruction of multi-DAT complexes responsible for the effect of tolerance and decreased cocaine seeking. Therefore, deconstruction of these complexes could be used for treatment of cocaine addiction [271]. Martin and Naughton have shown that DAT inhibition induces an increase in the dendritic spine density in the NAc [272]. Consequently, DAT-inhibition may have an effect on the long-lasting morphological changes in neurons associated with cocaine exposition and drug addiction.

#### 7.2.1. Glu Transporters

Apart from the receptors, Glu transporters also play an important role in the efficiency of synaptic transmission in NAc [273]. Their function relies on adjustment of the level of synaptic excitation/inhibition in response to different types of stimuli, duration of this effect, and its synchronization in different parts of NAc or in different subpopulations of projection neurons [273]. Natural reward and pain have different effects on expression of the vesicular glutamate transporters (VGLUTs) in NAc, which is related to specific types of excitation exerted by these stimuli [274]. For example, these differences are reflected by the expression level of VGLUT type 3 (VGLUT3) and VGLUT type 1 (VGLUT1) transporters in NAc after applying a natural reward or chronic pain. Sucrose consumption has been reported to increase only VGLUT3 expression, while chronic pain leads to a decrease in both VGLUT3 and VGLUT1 [73]. Interesting preliminary results regarding the function of glutamate transporters in regulation of physiological processes occurring in NAc and other BRS structures justify further research to clarify their role in addiction and CNS diseases.

#### 7.2.2. GABA Transporters

Activity of GABA-ergic neurons and release of GABA have a significant impact on the activity of projection neurons and, thus, indirectly on the concentration of DA in NAc [275]. This process is controlled by the plasma membrane GABA uptake transporters (GATs) located on astrocytes and neurons. It should be emphasized that there are differences in the mechanisms controlling DA release in the dorsal striatum and the NAc core resulting from different concentration of GAT transporters [275]. In the former, its release is controlled primarily by GAT-1 and GAT-3 transporters, which are more numerous in this area than in the NAc core.

Expression level of vesicular GABA transporter (vGAT) and vesicular glutamate transporter 2 (vGlut2) in NAc of the adolescent and adult rats after ethanol exposure shows a characteristic ontogenetic pattern with lower vGlut2/vGAT ratios in adolescents compared to adults [276]. The presented data suggest the constantly changing influence of excitatory (glutaminergic) and inhibitory (GABA-ergic) projections on NAc at various stages of ontogenetic development.

As shown by studies performed in mouse experimental models, chronic unpredictable mild stress is often associated with development of major depression [277]. During this process, the GABA-ergic neurons in NAc are damaged. One of the consequences of this damage, apart from a decrease in GABA release, is also a reduction in the levels of vesicular GABA transporters [277]. The presented results of the studies conducted so far indicate, on the one hand, the diversified role of GABA-ergic transporters in a number of regulatory processes in NAc. On the other hand, they show our still fragmentary knowledge about the meaning of these processes and underline the need for further research.

#### 7.2.3. 5-HT Transporter (SERT)

Results of a recent study have shown that SERT deletion contributes to the protection against the development of behavioral sensitization by increasing serotonergic neurotransmission, which is accompanied by dendritic remodeling of the MSNs in NAc [278]. These results could be useful for better understanding the evolution of changes in the course of addiction to methamphetamine and other psychotropic agents [278]. Other studies have shown that reduced expression of SERT could be a risk factor for development of cocaine addiction [279]. According to some authors, molecular, cellular and behavioral changes induced by cocaine abuse are the result of simultaneous modulation of the DA, NE and 5-HT transporters function [280]. Inhibition of these transporters may favor the development of morphological changes in the form of an increased density of dendritic spines in the NAc neurons [272]. These changes are an important element in the development of drug addiction.

#### 7.2.4. NE Transporter

Results of the study by Verheij and Karel have shown that changes in NE content do not have a decisive influence on the enhancement of cocaine intake in the SERT knockout rats [279]. In the NAc shell, as well as other brain regions, DA reuptake occurs by NE terminals [281]. In the NAc shell of the DAT knock-out mice exposed to cocaine, DA level can also be raised due to preventing its uptake by the NE transporter [281,282]. This phenomenon explains the causes of psychostimulant addiction. Moreover, antidepressants that bind selectively the NE transporter exert their therapeutic effect by increasing concentration of both DA and NE [281].

#### 7.2.5. DAT and SERT Knock-Out Models in Studies on NAc and BRS

Studies on the mechanisms of reward, stress, depression and addiction involve animal knock-out models of serotonin and dopamine transporters (SERT ko and DAT ko, respectively) [283,284,285,286]. Taking into account the fact that serotonin (SERT) plays a significant role in modulation of Glu neurotransmission in many brain areas, attention was drawn to this relationship occurring in NAc in the cocaine addiction [146]. In the SERT ko mouse model, effects of the reduced 5-HT content in both naïve mice and these previously exposed to cocaine were investigated [146]. In the cocaine-naïve mice, SERT deletion induced a reduction of Glu signaling leading to a decrease in its transmission efficiency. This is confirmed by the reduced expression of vGLUT1 and GLT-1 transporters which is related to the release and clearance of Glu from the synaptic cleft, respectively. In addition, there is a decrease in expression of the NMDA and AMPA receptor subunits. Overall, these changes suggest their adaptive character, resulting from the reduced glutamatergic transmission and lack of the modulating effect in mice with SERT deletion. This may explain the cocaine seeking behavior and increased self-administration observed in the mice with SERT deletion [287,288,289]. On the other hand, in rats exposed to cocaine for a long time, an increase in the content of vGLUT1 and GLT-1 was observed together with up-regulation of NMDA and AMPA receptor subunits [146]. These changes may be explained by sensitizing of the glutamatergic synapses during the long-lasting cocaine access.

The dopamine transporter (DAT) is responsible for removing the neurotransmitter from the synaptic cleft to the presynaptic terminal. The DAT ko mice show symptoms resulting from the increased DA content [284]. The use of DAT ko allows the evaluation of the biochemical and behavioral effects of an increase in DA concentration in the structures of the BRS [290]. DAT ko could be a useful model in studies on interaction mechanisms between psychostimulants and addictive drugs, such as amphetamine and cocaine, also taking into account the role of transporters for other neurotransmitters, such as 5-HT and NE [284,285].

### 7.3. Brain-Derived Neurotrophic Factor

Some authors emphasize the importance of brain-derived neurotrophic factor (BDNF) for proper functioning of connections between structures of the BRS, such as VTA, NAc, and PFC [291,292,293]. The specific role of BDNF in reward mechanisms results, among other factors, from its action on the VTA dopaminergic neurons projecting to NAc. BDNF plays an important role in synaptic plasticity [294]. There is well-documented data on the role of BDNF in LTP in several brain areas [295]. However, it should be emphasized that BDNF activity in this process is largely dependent on the brain region, nature of the stimulus, and age. The results of animal studies have shown that the expression levels of BDNF and the TrkB receptor differ between young and adult individuals [296]. Moreover, there are region-specific differences in BDNF expression between young and old individuals [296]. In general, while in NAc the BDNF expression is higher in adolescence than in adulthood, the opposite effect is observed in the PFC. The diversity of BDNF expression in the specific age groups, on the one hand, reflects the role of this neurotrophic factor in the processes of brain maturation associated with the development of learning, memory and elaboration of behavioral responses under the influence of natural stimuli [296]. On the other hand, it may be related to differences between the specific age groups in terms of susceptibility to drug-induced addiction and certain mental illnesses.

The relationship between changes in BDNF expression and altered functioning of the limbic system’s structures (e.g., PFC, NAc, Hip, Amg) has been documented in several mental disorders such as depression, schizophrenia, and drug-induced addiction [42,297,298,299]. In summary, BDNF plays an important role in the regulation of synaptic plasticity in the BRS. Concentration of this neurotrophic factor varies depending on age, brain area, and type of stimulus.

## 8. Conclusions

Data presented in this review show a wide range of NAc functions, not only under physiological conditions but also in pathological processes. The unique role of NAc among the structures of the BRS is a consequence of: (1) a widely distributed hierarchical system of connections with other brain regions, (2) cooperation with the limbic and motor functional systems in regulating the state of consciousness and behavioral reactions, and with the vegetative system, in coordinating metabolic, endocrine, and autonomic nervous systems functions, (3) cooperation among several neurotransmitter systems, (4) well-developed morphological and functional plasticity processes enabling control of the short- and long-term synaptic enhancement, and (5) supportive role of the NAc neuroglia in physiological and pathological processes.

Changes in NAc functioning contribute to the development of several CNS diseases, such as depression, schizophrenia, and AD. In all these cases, the NAc dysfunction should be analysed in the context of its hierarchical connections with the other CNS structures and functional systems, impairment of the neurotransmitter systems and neuroplasticity processes. Further research on the structure and function of NAc will provide relevant information, useful not only for a better understanding of the mechanisms regulating motivation processes and striving for reward-achievement but possibly also for the development of effective therapies for some CNS diseases.

## Data Availability

Not applicable.

## Abbreviations

α1AR	α1-adrenergic receptor
α2AR	α2-adrenergic receptor
Amg	amygdala
AmgBL	basolateral part of amygdala
Amgex	extended amygdala
AMPARs	α-amino-3-hydroxy-5-methyl-4-isoxazolepropionic acid-type glutamate receptors
BDNF	brain-derived neurotrophic factor
BF	basal forebrain
BRS	brain reward system
CDn	caudate nucleus
cAMP	cyclic adenosine monophosphate
CREB	cAMP-response element-binding protein
DA	dopamine
dHip	dorsal hippocampus
dmPFC	dorso-medial prefrontal cortex
D1R	D1 dopamine receptor
D2R	D2 dopamine receptor
DRn	dorsal raphe nucleus
ERK	extracellular signal-regulated kinase
GABA	γ-aminobutyric acid
GDP	guanosine diphosphate
Glu	glutamate
GP	globus pallidus
GPex	external globus pallidus
GPin	internal globus pallidus
GTP	guanosine triphosphate
Hip	hippocampus
5-HT	serotonin
HTh	hypothalamus
IFN-γ	interferon gamma
IL-1β	interleukin-1 beta
IThn	intralaminar thalamic nuclei
LC	locus coeruleus
LTP	long-term potentiation
LHn	lateral habenular nucleus
LHTh	lateral hypothalamus
LPa	lateral preoptic area
LV	lateral ventricle
MBP	myelin basic protein
Mctx	motor cortex
MDD	major depressive disorder
MDThn	mediodorsal thalamic nucleus
mloPFC	medial and lateral orbital prefrontal cortex
MOBP	myelin-associated oligodendrocyte basic protein
mPFC	medial prefrontal cortex
MThn	midline thalamic nuclei
NAc	nucleus accumbens
NAcc	core of nucleus accumbens
NAcs	shell of nucleus accumbens
NE	norepinephrine
NF-κB	nuclear factor-kappa B
NPAS2	neuronal PAS domain 2 protein
PFC	prefrontal cortex
PHpg	para-hippocampal gyrus
PLP1	proteolipid protein 1
PPn	pedunculopontine nucleus
preMctx	premotor cortex
PSD95	postsynaptic density of protein 95
Put	putamen
SNpc	substantia nigra pars compacta
SNpr	substantia nigra pars reticulate
Spt	septum
Sub	subiculum
TegB	bulbar tegmentum
Th	thalamus
TrkB	tyrosine receptor kinase B
VGLUT	vesicular glutamate transporter
vHip	ventral hippocampus
VP	ventral pallidum
VTA	ventral tegmental area
